# Uniaxial transition dipole moments in semiconductor quantum rings caused by broken rotational symmetry

**DOI:** 10.1038/s41467-019-11225-6

**Published:** 2019-07-22

**Authors:** Nicolai F. Hartmann, Matthew Otten, Igor Fedin, Dmitri Talapin, Moritz Cygorek, Pawel Hawrylak, Marek Korkusinski, Stephen Gray, Achim Hartschuh, Xuedan Ma

**Affiliations:** 1 Department of Chemistry and Center for NanoScience (CeNS), LMU Munich, 81377 Munich, Germany; 2 Center for Nanoscale Materials, Argonne National Laboratory, Argonne, IL 60439 USA; 3 Department of Chemistry and James Franck Institute, University of Chicago, Chicago, IL 60637 USA; 4 Department of Physics, University of Ottawa, Ottawa, ON K1N 6N5 Canada; 5 Quantum Theory Group, Security and Disruptive Technologies, National Research Council, Ottawa, K1A0R6 Canada

**Keywords:** Nanoscale materials, Quantum optics

## Abstract

Semiconductor quantum rings are topological structures that support fascinating phenomena such as the Aharonov–Bohm effect and persistent current, which are of high relevance in the research of quantum information devices. The annular shape of quantum rings distinguishes them from other low-dimensional materials, and enables topologically induced properties such as geometry-dependent spin manipulation and emission. While optical transition dipole moments (TDMs) in zero to two-dimensional optical emitters have been well investigated, those in quantum rings remain obscure despite their utmost relevance to the quantum photonic applications of quantum rings. Here, we study the dimensionality and orientation of TDMs in CdSe quantum rings. In contrast to those in other two-dimensional optical emitters, we find that TDMs in CdSe quantum rings show a peculiar in-plane linear distribution. Our theoretical modeling reveals that this uniaxial TDM originates from broken rotational symmetry in the quantum ring geometries.

## Abstract (web summary)

Annular semiconductor structures, or “quantum rings”, are of interest for quantum information and photonics applications. Here, the authors show that breaking rotational symmetry through elongation generates an in-plane optical transition dipole moment in CdSe quantum rings.

## Introduction

Optical transition dipole moments are key parameters in determining the interactions between optical emitters and external electromagnetic fields. Controlled orientation and alignment of TDMs have led to optoelectronic^1,2^ and quantum photonic^3,4^ devices with unprecedented performance. The properties of TDMs are determined by the emitters’ electronic structures, which are closely related to their compositions, dimensions, and geometries. For instance, TDMs in zero-dimensional semiconductor quantum dots and two-dimensional nanoplatelets and transition-metal dichalcogenides have shown anisotropic two-dimensional distributions^5–8^, whereas those in one-dimensional nanorods and nanowires are uniaxial^9,10^.

In semiconductor quantum rings (QRs), charge carriers are confined in the radial direction. This quantum confinement effect, in combination with the unique ring geometry, has led to the observation of geometry-dependent photon antibunching^11^ and angular momentum-dependent emission in QRs^12^, which may enable a new class of quantum photon sources capable of high-density information coding for quantum communication^13,14^ and quantum imaging techniques^15^. The Aharonov-Bohm effect in QRs^16,17^ may also provide a promising avenue for single photon trapping, storage, and release by the use of an electric field^18^. To explore these intriguing quantum photonic properties of QRs, a critical first step required for their realization and application in quantum information science is a detailed understanding of their intrinsic TDMs. Moreover, the study of TDMs in a topological ring structure is fundamentally interesting as QRs represent a crossover between one- and two-dimensional structures due to their peculiar annular geometry.

Here, we investigate the dimensionality and orientation of the TDMs in CdSe QRs using single particle optical spectroscopy. We find that despite their two-dimensional annular shape, CdSe QRs exhibit an in-plane linear distribution. Our empirical tight binding calculations show that this uniaxial TDM is caused by the broken rotational symmetry in the QR geometries.

## Results

### Quantum ring geometry and optical spectra

**Fig. 1:**
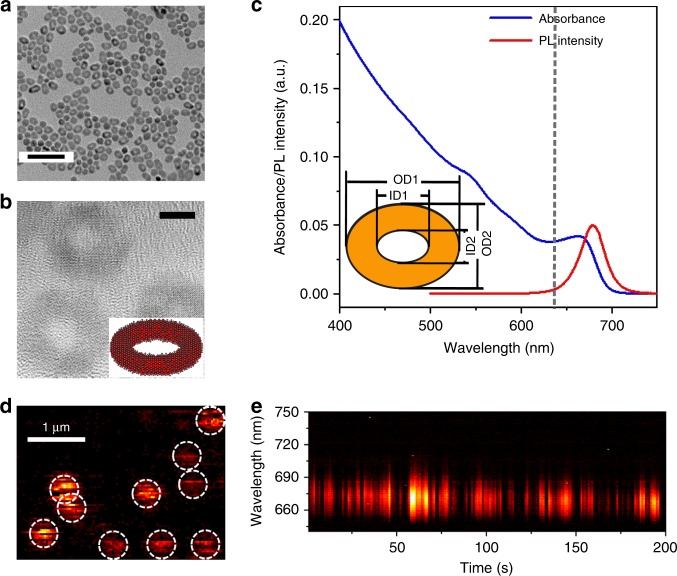
Ensemble and single particle characterization of CdSe quantum rings. **a**, **b** TEM images of the CdSe QRs. Scale bars in **a** and **b** represent 50 nm and 5 nm, respectively. **c** Absorption and emission spectra of CdSe QR ensembles. The vertical dashed line indicates the excitation wavelength for the single particle measurements. The inset is a schematic of the ring geometry. **d** A scanning photoluminescence image of single QRs. Scale bar: 1 µm. **e** A representative time-dependent spectral sequence of a single QR

CdSe QRs with emission wavelengths at around 675 nm (Fig. 1a–c) were synthesized following our previously published method^19^. Their average outer ring diameters (OD_1_ and OD_2_) along two perpendicular axes are 13.3 ± 1.1 nm and 9.9 ± 1.0 nm, respectively, with certain degrees of lateral elongation visible (see Supplementary Note 1 for statistics). The corresponding average inner ring diameters, ID_1_ and ID_2_, are 6.1 ± 0.9 nm and 3.8 ± 0.7 nm, respectively. Hence, the average thickness of the rings varies from *t*_1_ = 3.6 nm along the long axis to *t*_2_ = 3.0 nm along the short axis. The average height *h* of the QRs was around 5.0 nm. Emissive QRs dispersed on substrates were located by raster scanning samples through the focus of a laser beam. Time-dependent measurements of single QR spectra show photoluminescence blinking and spectral shift (Fig. 1e), both of which are characteristic of single quantum emitters and often attributed to charge carrier trapping at surface states^20–22^.

### Angle-resolved photoluminescence spectroscopy

**Fig. 2:**
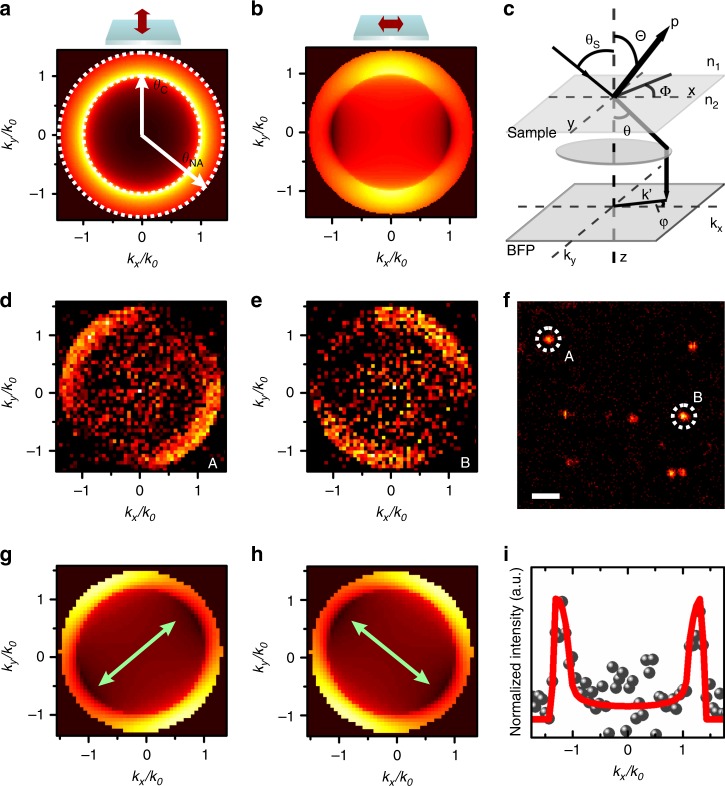
**k**-vector resolved Fourier imaging of single CdSe quantum rings. **a**, **b** Calculated **k**-vector resolved emission pattern of a TDM lying vertically **a** and horizontally **b** on a sample plane. The critical angle (*θ*_C_) and the angle determined by the numerical aperture (*θ*_NA_) are indicated. **c** Schematic of angle-resolved photoluminescence spectroscopy on a Fourier imaging system. **d**, **e** Measured radiation patterns of QRs highlighted in **f**. **f** A scanning photoluminescence image used to locate single QRs. Scale bar: 2 µm. **g**, **h** Calculated radiation patterns of QRs with their azimuthal angles adjusted to match those in **d** and **e**. **i** **k**-vector resolved emission profiles from experimentally measured (dots) and calculated (curve) radiation patterns

Since the interaction strength of optical emitters with light is indicative of their intrinsic TDMs, we apply two independent imaging approaches to reveal the TDMs of the CdSe QRs. In both approaches, isolated CdSe QRs with random azimuthal orientations are dispersed on substrates and loaded onto home-built confocal laser microscopes. We excite the CdSe QRs at a wavelength of 633 nm to ensure that only band-edge optical transitions are involved. We first investigate the QRs utilizing angle-resolved photoluminescence spectroscopy on a Fourier imaging system (Fig. 2c. See Supplementary Note 2 for a detailed description of the setup). This technique allows direct imaging of **k**-vector resolved emission projections containing information about the orientations of the TDMs^23,24^. It is essential that a high numerical aperture (NA = 1.4) objective is used to focus the laser beam onto a single QR at a time and collect the corresponding emission. Intensity distributions of the QR emission at the back focal plane of the objective are recorded by a charge-coupled device. Our numerical simulations (see Supplementary Note 2 for details) show that the **k**-vector resolved emission pattern of a uniaxial TDM lying horizontally on a sample surface forms two bright lobes, whereas that of a uniaxial TDM lying vertically to the sample surface form a circular ring with an intensity nearly isotropic in the azimuthal angle (Fig. 2a, b). The distinct boundary between an inner and an outer region in the momentum space corresponds to the critical angle (*θ*_C_) of total internal reflection at the glass-air interface, and radiation emits predominantly at angles larger than the critical angle into the high refractive index medium. The outer circle is defined by the maximum collection angle of the objective used in the measurements (*θ*_NA_). Figure 2d, e show representative **k**-vector resolved radiation patterns measured from single CdSe QRs. Calculated radiation patterns of in-plane uniaxial dipoles with their azimuthal orientations adjusted to match the experimentally measured patterns are shown in Fig. 2g, h. Good agreement between the experimentally measured and theoretically simulated radiation patterns can be observed. This is further confirmed by comparing the **k**-vector resolved emission profile cuts from the experiments and calculations (Fig. 2i). Additional **k**-vector resolved cross sections parallel and perpendicular to the dipole axes shown in Fig. 2d, e are presented in Supplementary Note 2, and they show good agreement with the simulated profiles extracted from Fig. 2g, h. This infers that the TDMs in CdSe QRs are in-plane and uniaxial.

### Higher-order laser scanning microscopy

**Fig. 3:**
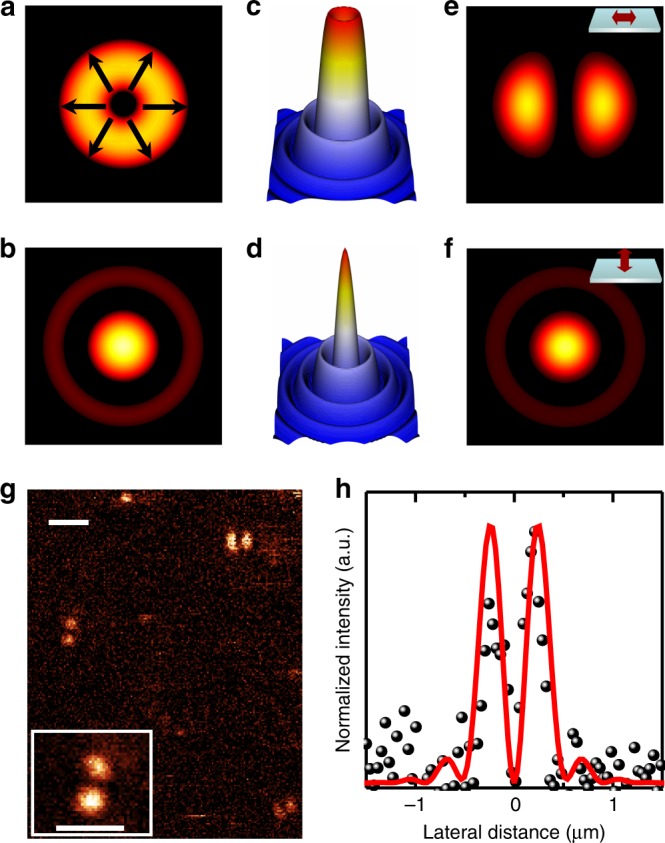
Higher-order laser mode scanning microscopy of single CdSe quantum rings. **a**, **b** Numerically simulated transverse and longitudinal components of a radially polarized Bessel-Gauss laser beam. **c, d** The corresponding intensity profiles of the transverse **c** and longitudinal **d** electromagnetic components. **e, f** Calculated excitation patterns of a uniaxial TDM lying horizontally **e** or vertically **f** on the sample plane. **g** Experimentally measured excitation patterns of individual CdSe QRs. **h** Calculated intensity profiles of a horizontally lying uniaxial TDM (curve) and measured intensity profiles from the excitation patterns of a single CdSe QR (dot). The dimensions of **a**, **b**, **e**, **f** are 1.27 × 1.27 µm^2^. The dimensions of **c**, **d** are 2.53 × 2.53 µm^2^. The scale bars in **g** are 1 µm

To consolidate our conclusion of the uniaxial, in-plane nature of the TDMs in CdSe QRs, we apply a second approach based on higher-order laser scanning microscopy to study the absorption in QRs. This approach has been used to study TDMs of organic molecules^25^, and semiconductor^8,26^ and metallic nanoparticles^27^. When an individual particle is raster scanned through the focus of a higher-order Bessel–Gauss laser beam, its excitation pattern is characteristic of its TDMs at the excitation wavelength. Figure 3a, b shows the calculated transverse and longitudinal field components within the focal region of a radially polarized laser beam according to the parameters used in our experiment. The corresponding field intensity profiles are shown in Fig. 3c, d. When a uniaxial TDM lies horizontally in the sample plane, it interacts mainly with the transverse component of the radially polarized laser beam, leading to an excitation pattern consisting of two bright off-axis lobes (Fig. 3e). In the case that the uniaxial dipole lies perpendicularly to the sample plane, primarily the longitudinal component of the radially polarized laser beam interacts with the dipole, giving rise to an intense spot in the center. The relatively weak interaction between the transverse component of the laser beam and the vertical dipole results in a weak ring around the intense spot (Fig. 3f). Comparison between the calculated and experimentally measured excitation patterns enables the determination of the dimensionalities and orientations of the TDMs. Excitation patterns of individual CdSe QRs scanned through radially polarized laser beams exclusively show two bright off-axis lobes (Fig. 3g). The intensity profiles from the experimentally measured patterns and calculated patterns of a horizontally lying uniaxial dipole show a reasonable agreement (Fig. 3h). These results confirm our conclusion about the uniaxial, in-plane nature of the TDMs in CdSe QRs. We note that the TDMs studied here are the intrinsic properties of the QRs given their specific composition and geometry, while their emission polarization may not be uniaxial due to the renormalization effect caused by the differences in the dielectric constants of the QRs and their surrounding environment^8,28,29^.

### Calculations of wavefunctions

**Fig. 4:**
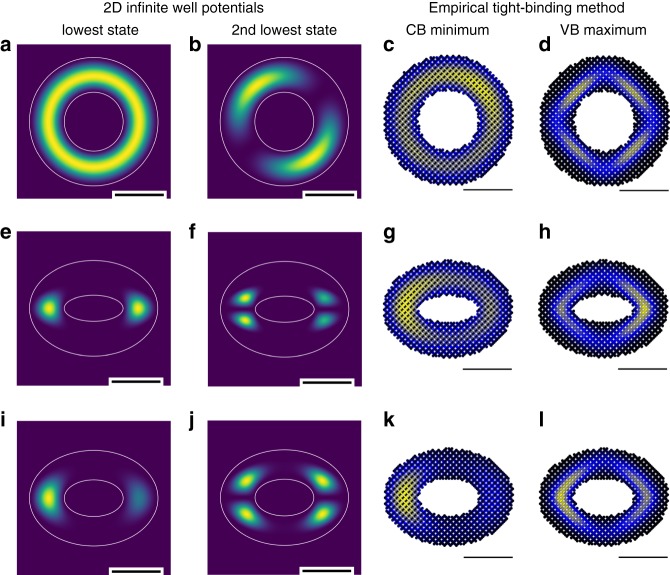
Calculations of the electronic structures of CdSe quantum rings. **a**, **b** Calculated wavefunctions of the two lowest states of two-dimensional infinite well potentials with OD_1_ = OD_2_ = 13.3 nm, ID_1_ = ID_2_ = 6.1 nm, and *t*_1_ = *t*_2_ = 3.6 nm. **c**, **d** 2D slices of wavefunctions of a CdSe QR (OD_1_ = OD_2_ = 13.3 nm, ID_1_ = ID_2_ = 6.1 nm, *t*_1_ = *t*_2_ = 3.6 nm, and *h* = 5.0 nm) corresponding to the conduction band (CB) minimum **c** and the valence band (VB) maximum **d** calculated using an empirical tight-binding method. The slices are in the *xy* plane and cut through the center of the rings. **e**, **f** Calculated wavefunctions of the two lowest states of two-dimensional infinite ring well potentials with OD_1_ = 13.3 nm, OD_2_ = 9.9 nm, and ID_1_ = 6.1 nm, ID_2_ = 2.7 nm, and *t*_1_ = *t*_2_ = 3.6 nm. Breaking of the continuous rotational symmetry leads to the lifting of the *p*- and *d*- type orbitals as the two lowest states. An optical transition between these two orbitals has a uniaxial transition dipole. **g**, **h** 2D slices of wavefunctions of a CdSe QR (OD_1_ = 13.3 nm, OD_2_ = 9.9 nm, and ID_1_ = 6.1 nm, ID_2_ = 2.7 nm, *t*_1_ = *t*_2_ = 3.6 nm, and *h* = 5.0 nm) corresponding to the conduction band minimum **g** and the valence band maximum **h** calculated using an empirical tight-binding method. **i**, **j** Wavefunctions of the two lowest states of two-dimensional infinite ring well potentials with OD_1_ = 13.3 nm, OD_2_ = 9.9 nm, ID_1_ = 6.1 nm, ID_2_ = 3.8 nm, *t*_1_ = 3.6 nm, and *t*_2_ = 3.0 nm. **k**, **l** 2D slices of wavefunctions of a CdSe QR (OD_1_ = 13.3 nm, OD_2_ = 9.9 nm, and ID_1_ = 6.1 nm, ID_2_ = 3.8 nm, *t*_1_ = 3.6 nm, *t*_2_ = 3.0 nm, and *h* = 5.0 nm) corresponding to the conduction band minimum **k** and the valence band maximum **l** calculated using an empirical tight-binding method. Localization of the band edge states due to broken rotational symmetry can be observed. Scale bars: 5 nm

In bulk zinc blend CdSe, the heavy-hole and light-hole bands are degenerate. However, in a quasi-two-dimensional zinc blend CdSe nanoplatelet, the quantum confinement in the *z* ([001]) direction leads to a lifted heavy-hole band which possesses a mixed *p*_*x*_ and *p*_*y*_ symmetry with no *p*_*z*_ contributions^7^. Here, *x* and *y* are in-plane directions. Thus, an optical transition between the conduction band edge state, which has a *s*-like symmetry, and heavy-hole band forms an isotropically distributed in-plane TDM. CdSe QRs could be considered as two-dimensional topological structures that are warped analogs of quantum wires. However, in stark contrast to CdSe nanoplatelets^7,8^ and other two-dimensional optical emitters such as transition-metal dichalcogenides^6^ which possess isotropic two-dimensional TDMs, QRs are found here to show TDMs with properties similar to that of one-dimensional structures^9^. It appears that topology plays a significant role in determining their TDMs. To understand this finding, we calculate the electronic energy levels and wavefunctions of the CdSe QRs that determine their TDMs. We start by looking at the solutions of two-dimensional infinite ring well potentials. The potentials are defined by the ring outer diameters along the *x* and *y* axes to the center of the ring (OD_1_ and OD_2_) and the corresponding inner diameters (ID_1_ and ID_2_). The ring thicknesses *t*_1_ and *t*_2_ along the *x* and *y* axes are defined as: *t*_1_ = $\frac{1}{2}$(OD_1_−ID_1_) and *t*_2_ = $\frac{1}{2}$(OD_2_−ID_2_). Within the ring, the potential is set to zero, whereas outside of the ring, the potential is infinite. When OD_1_ = OD_2_ = 13.3 nm and *t*_1_ = *t*_2_ = 3.6 nm (Fig. 4a, b), the ring is circular with a continuous rotational symmetry. We use the discrete variable representation to solve this system^30^. Solutions to the Schrödinger equation with this potential admit an *s*-type orbital, where the wavefunction shows a symmetric distribution through the ring, as the ground state, and *p*-type orbitals, where there are two lobes along different axes, as the second lowest state. Due to the continuous rotational symmetry, these solutions are not unique. A rotation of any amount of the *p*-type orbitals would also be a solution. However, when one of the axes is longer than the other (OD_1_ = 13.3 nm, OD_2_ = 9.9 nm) while the ring thickness is kept constant (*t*_1_ = *t*_2_ = 3.6 nm), breaking of the continuous rotational symmetry leads to the lifting of the *p*- and *d*-type orbitals as the two lowest states, which now show a preferential location for the wavefunction lobes (Fig. 4e, f). In this case, there is a preferred direction for a uniaxial dipole excitation between the two. Further adoption of the experimental geometry of OD_1_ = 13.3 nm, OD_2_ = 9.9 nm, and *t*_1_ = 3.6 nm, *t*_2_ = 3.0 nm gives similar wavefunction distributions and uniaxial dipole orientations (Fig. 4i, j).

To simulate the QR structures with realistic potentials, we solve for the band-edge states using an empirical tight-binding method utilizing the QNANO computational platform (see Methods for details)^31,32^. Since we focus on band edge single particle energy states, we do not directly take into account exchange splitting. However, we expect this effect to be small as magnitudes of anisotropic exchange splitting in CdSe nanostructures^33–35^ is much smaller than the thermal energies in our experiments. These calculations show that in realistic QRs, though the pure geometry is still a factor, a three-dimensional, atomistic ring adds additional complexity to the electronic structure. This is because the discrete nature of the atomic positions breaks the continuous rotational symmetry present in the infinite ring potential, even in the case of OD_1_ = OD_2_ = 13.3 nm, *t*_1_ = *t*_2_ = 3.6 nm, and *h* = 5.0 nm (Fig. 4c, d) (see Supplementary Note 3 for vertical cross sections). Here, the wavefunction of the lowest conduction band level (Fig. 4c) is no longer perfectly symmetric as was observed for the infinite well potentials, although it is still generally delocalized around the ring and keeps the *s*-type character. The overlap of the electron (Fig. 4c) and hole (Fig. 4d) wavefunctions results in a two-dimensional optical transition dipole moment with components along both the *x* and *y* axes. For CdSe QRs with OD_1_ = 13.3 nm, OD_2_ = 9.9 nm, *t*_1_ = *t*_2_ = 3.6 nm, and *h* = 5.0 nm (Fig. 4g, h), both the oval nature of the quantum ring and the atomic structure break the rotational symmetry. In this case, the elongation of the QR leads to the localization of wavefunctions in the thick end of the oval-shaped rings for both band edge states (Fig. 4g, h), although the wavefunctions are still mostly delocalized around the whole ring, and hence has a two-dimensional transition dipole. There are still some effects from the local atomic structures, which break the symmetry between the left and right lobes, although the effects are less significant compared to these in QRs with a smaller thickness (see Supplementary Note 4 and 5 for ring thickness dependent atomic structure effects). For the ring geometry that most closely represents our experimentally measured QR samples, the differential thickness (*t*_1_ = 3.6 nm, *t*_2_ = 3.0 nm) further breaks the rotational symmetry and causes additional localization of the wavefunctions in the thick end of the ring, as shown in Fig. 4k, l. This strong, geometry-induced localization leads to a uniaxial, in-plane dipole, as was observed in our experimental measurements. We note that even though the rings vary slightly in size and shape, they are essentially ovals with certain thickness variations (Supplementary Note 1). As shown in Fig. 4k, l and Supplementary Note 3–7, this geometry, over a wide range of possible parameters, gives rise to mostly uniaxial dipoles. Our simulations also show that the specific symmetry breaking between the different wavefunction lobes varies for QRs with various crystallographic orientations (Supplementary Note 6), but their TDMs still remain uniaxial and in-plane. Moreover, the calculated energy difference between the two band edge states of 1.84 eV matches very well with the experimentally observed band edge absorption and emission wavelengths (Fig. 1c).

## Discussion

In summary, we find that the symmetry-related electronic properties of nanostructures are highly susceptible to both geometrical variations and the local atomic structures. In our QR structure, the combination of these two effects leads to a localization of the wavefunctions and consequently, a uniaxial transition dipole moment in a two-dimensional QR structure. Our finding not only has important implications for the quantum photonic applications of QRs^11,17,18^, but may also enable the development of a new class of quantum materials. Specifically, the introduction of optical spin-orbit coupling through an asymmetric ring geometry may enable the occurrence of an optical Berry phase for photon manipulation and the examination of non-trivial topological effects^36^. The spatial asymmetry-caused Rashba effect could be utilized to obtain a long spin coherence time^37^. These features, together with the high spin stability in QRs^38^, make them attractive as spin qubits and for applications in spin field-effect transistors.

## Methods

### Synthesis of CdSe quantum rings

CdSe QRs were synthesized following a previously published method^19^. CdSe nanoplatelets were first synthesized at 240 °C from Cd myristate, Cd acetate, and Se power in 1-octadecene. They were then precipitated with ethanol and re-dispersed in a mixture of 1-octadecene and oleylamine in a three-neck flask. A suspension of Se in oleylamine was added into the flask as well. The contents of the flask were then degassed at room temperature for 10 min, heated up to 140 °C under nitrogen and maintained in this condition for 10 min. After injecting a certain amount of tributylphosphine into the flask, the temperature of the mixture was increased to 220 °C. The solution was then allowed to cool to 37 °C. Finally, ground Cd formate was added to the mixture and it was stirred for an hour.

Transmission electron microscopy of the samples were performed using a 300 kV FEI Tecnai F30 microscope. Absorption and emission spectra of the solutions were collected using a Cary 5000 UV-Vis-NIR spectrometer and FluoroMax-4 spectrofluoremeter, respectively.

### Single quantum ring optical spectroscopy and imaging

To prepare samples for single particle optical measurements, the stock solutions of QRs were diluted by hexane and spin coated onto quartz or glass cover slips. Optical measurements of single QRs were performed on home-built confocal laser scanning microscopes at room temperature. The angle-resolved photoluminescence spectroscopy was performed on a Fourier imaging system (see Supplementary Note 2). In this method, to inhibit photoluminescence blinking of the QRs, a layer of polystyrene was deposited onto the QRs. This was considered within the dipole distribution simulations by adjusting the refractive index of the upper half-space to *n*_1_ = 1.1, since the presence of the polystyrene thin film will increase the effective refractive index experienced by the QRs. The lower half-space refractive index *n*_2_ was defined by the glass cover slide, index matching oil and objective and given with *n*_2_ = 1.52. A continuous wave HeNe laser with a wavelength of 633 nm was focused by a microscope objective (NA = 1.4) to excite the samples. Emission from the QRs was collected by the same objective and sent to a cooled charge-coupled device (CCD) after spectral filtering. A Bertrand lens was used to form an image of the back focal plane on the CCD. The higher-order laser scanning microscopy of the QRs were performed using similar excitation conditions, except that the Gaussian laser beam was converted into a higher-order radially polarized beam and a microscope objective with NA = 1.49 was used. Emission from the QRs collected by the objective was focused onto an avalanche photon diode to form confocally scanned images.

### Theoretical modeling of quantum rings

The two-dimensional infinite potential well calculations were performed using a discrete variable representation with a sinc basis^30^. Two ovals, defined by the parameters OD_1_, OD_2_, ID_1_, and ID_2_ (see Fig. 1c), were defined. Any points in the discretized space in between these two ovals had a potential of zero; any point outside had a potential set arbitrarily high, defining a Hamiltonian matrix. This matrix was diagonalized using the parallel eigenvalue solver package SLEPc^39^ for the eigenstates with eigenvalues closest to zero.

To calculate the energy levels and wavefunctions of the CdSe QRs, we started with a large supercell of zinc-blend CdSe atoms with lattice constant 6.052 Å. We then defined an ideal three-dimensional generalized torus, where any slice along *z* was defined by two ovals in the *x*, *y* plane. In the middle of the ring along the *z* dimension, these two ovals were defined by the parameters OD_1_, OD_2_, ID_1_, and ID_2_. Towards the edges of the ring in the *z* plane (defined by the height of the ring, *h*), the parameters of the two ovals changed such that a slice along the *x* or *y* planes results in an oval. Any atom which fell inside of this three-dimensional geometry was kept; any atom outside of the ring was discarded. After cutting out the ring, we iteratively looped through all atoms and removed any atoms which had only a single bond until all atoms had at least two bonds. This may result in unequal numbers of Cd and Se in the QRs, but since we are not doing total energy calculations, a slight nonstoichiometry should not alter our calculation results. Using this set of atoms, we solved for the band-edge states using the empirical tight-binding method within the code QNANO^31,32^. Within the empirical tight-binding method, a set of orbitals was defined for each atom. The parameters of these orbitals were fit to density functional theory calculations of bulk systems. The method solved for the eigenstates near the band-edge using the basis of these parameterized orbitals.

## Supplementary information

Supplementary Information

## Data Availability

The data that support the findings of this study are available from the corresponding author upon reasonable request.
