# Supplementary material for: Uniaxial transition dipole moments in semiconductor quantum rings caused by broken rotational symmetry
Source: Nat Commun. 2019 Jul 22;10:3253. doi: 10.1038/s41467-019-11225-6 (PMC6646311; doi:10.1038/s41467-019-11225-6)
Supplement: Supplementary file 1 — Supplementary Information [file 41467_2019_11225_MOESM1_ESM.pdf]

Supplementary Information for:

Uniaxial Transition Dipole Moments in Semiconductor Quantum Rings Caused by Broken Rotational Symmetry

Hartmann *et al*.

Supplementary Note 1 - Quantum ring geometries

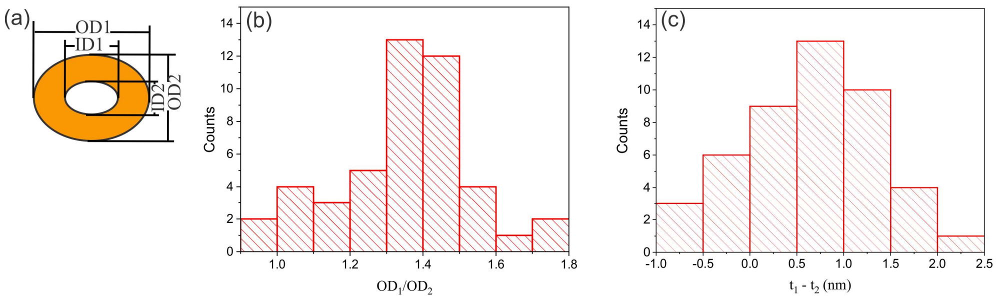

Supplementary Figure 1. (a) Schematic of the quantum ring geometry. (b) Histogram of the QR aspect ratios defined as the ratio between the outer ring diameters along the two orthogonal axes, OD_1/OD_2. Most of the rings show certain elongation and possess an oval shape. (c) Histogram of the QR thickness variation along the two orthogonal axes, $t_1 - t_2$. Majority of the rings show thickness variations.

Supplementary Note 2 - Experimental details and analytical calculations of the k-vector resolved emission patterns

The setup used for the recording of the **k**-vector resolved emission patters is based on an inverted confocal microscope (Supplementary Figure 2a). The excitation source is a linearly polarized 633 nm HeNe laser, which is reflected by a non-polarizing dichroic beam-splitter and focused onto the sample with a NA = 1.4 oil-immersion objective. PL from the quantum rings is collected by the same objective, transmitted by the beam-splitter, and after being separated from the remaining excitation light using a long-pass filter, recorded by a charge coupled device (CCD) image array. In order to record the non-polarization selective angular intensity distributions of the emitters, a Bertrand lens following an intermediate tube lens[1] was chosen. Together with the selected resolution of the CCD array this results in a **k**-space resolution k_x/k_0 and k_y/k_0 of about 0.0667 per image pixel.

The intensity distributions $I(k_x/k_0, k_y/k_0)$ in the back focal plane are calculated using the *p*- and *s*-polarized components of the electric fields radiated by a point dipole **p** on the air-glass interface depending on its in-plane orientation Φ and out off-plane orientation Θ, the polar emission angle φ and the distance from the center *r*:[2]

$$I(r, \varphi, \Theta, \Phi) = \frac{1}{cos\theta}\left(E_p E_p^* + E_s E_s^*\right). \qquad 1$$

Additional fixed parameters to describe the observed experimental configuration are the refractive indices n_1 and n_2 of the two half-spaces defining the interface as well as NA of the used microscope objective.

The emission pattern of a point-dipole oriented in the sample plane has a distinct form (Supplementary Figure 2). On the borders of the circle, the pattern has two half-moon shaped maxima on opposite sides. The line connecting the maxima is perpendicular to the real space orientation of the radiating dipole **p**. Therefore, the orientation of the emitting dipole can be determined from the orientation of the two maxima.

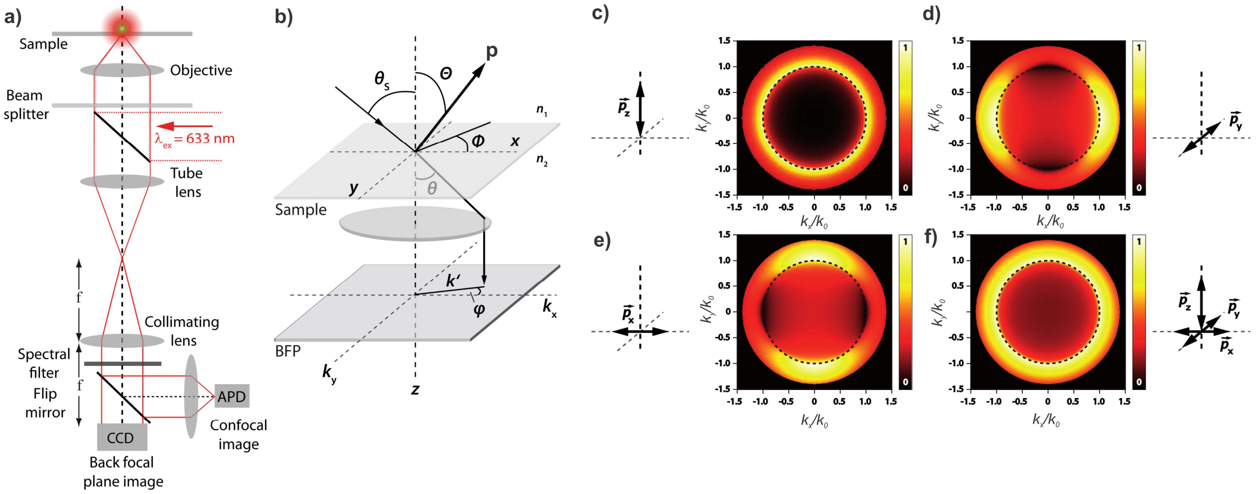

Supplementary Figure 2. (a) Optical microscope setup for detecting radiation patterns in the back focal plane. (b) Schematic of the dipole orientation and the coordinate system used in the calculation. (c) – (f) Numerically calculated radiation patterns for differently oriented point dipoles.

In order to reduce photoluminescence blinking of the quantum rings for the recording of the emission patterns, a polystyrene thin film was spin-coated on top of the dispersed sample on a glass cover-slide. This was considered within the calculation by adjusting the refractive index of the upper half-space to $n_1 = 1.1$, since the presence of the polystyrene thin film will increase the effective refractive index experienced by the QR emitter. The lower half-space refractive index n_2 is defined by the glass cover slide, index matching oil and objective and given with $n_2 = 1.52$.

To further demonstrate the in-plane uniaxial dipolar nature of the QR TDMs, additional cross-sections extracted from the emission patterns of the two representative QRs A and B in Fig. 2d-e of the main text are presented in Supplementary Fig. 3 and 4. Together shown are the corresponding simulated profiles extracted from Fig. 2g-h. Cross sections parallel to the dipole directions (Supplementary Fig. 3(c) and 4(c)) show a near flat response in the experiment and calculated data. The weak contribution for **k**-vectors larger than $k_{||}/k_0 = 1$ in the calculated patterns are not recognizable in the experiment due to the background noise. However, in the cuts perpendicular to the dipole directions (Supplementary Fig. 3(d) and 4(d)) the two maxima towards the outer area of the patterns are nicely reproduced in the experiment.

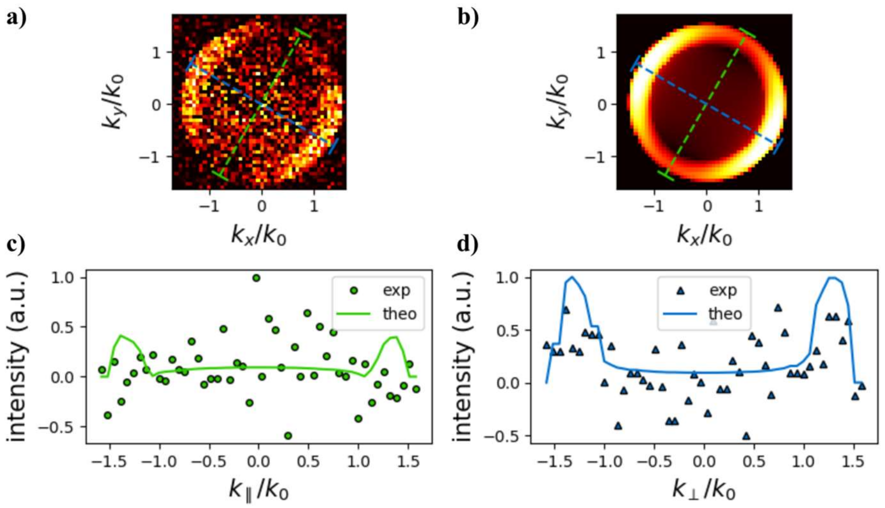

Supplementary Figure 3. Experimental (a) and calculated (b) emission patterns from the example QR A in Fig. 2 of the main text. The orthogonal cuts, parallel (c) and perpendicular (d) with respect to the dipole orientation, show good agreement between the experimental (green circles, blue triangles) and calculated values (green solid line, blue solid line).

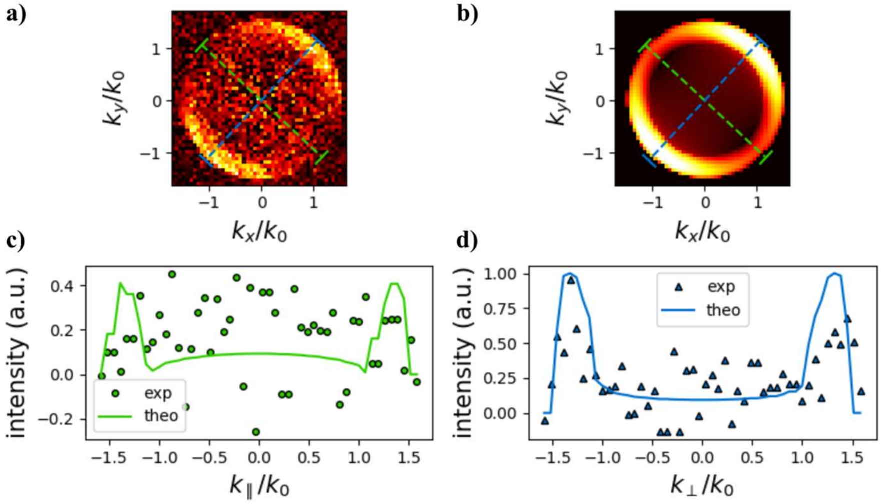

Supplementary Figure 4. Experimental (a) and calculated (b) emission patterns from the example QR B in Fig. 2 of the main text. The orthogonal cuts, parallel (c) and perpendicular (d) with respect to the dipole orientation, show good agreement between the experimental (green circles, blue triangles) and calculated values (green solid line, blue solid line).

Supplementary Note 3 - Wavefunctions of CdSe quantum rings in different cutting planes

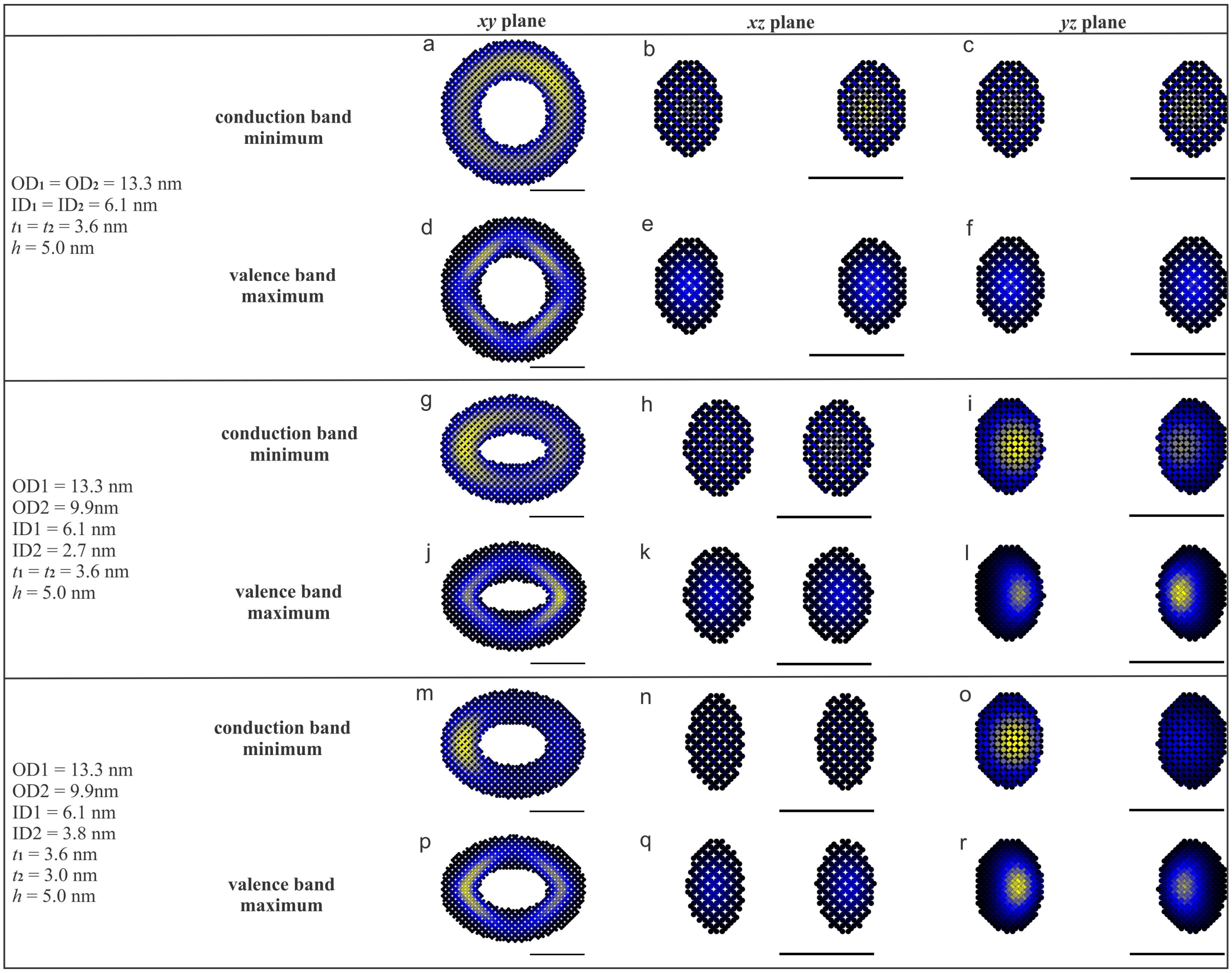

Supplementary Figure 5. 2D slices of wavefunctions of CdSe QRs with different geometries cut along different directions. All the slices go through the ring centers. Scale bars: 5 nm.

Supplementary Note 4 - Wavefunctions of thin CdSe quantum rings

We first look at two-dimensional infinite ring well potentials of a thin CdSe quantum well. The two outer and inner diameters of the ring are set to be equal to each other, $OD_1 = OD_2 = 13$ nm, $ID_1 = ID_2 = 9.6$ nm. In this case, there is a continuous rotational symmetry. The ground state has a *s*-type orbital, where the wavefunction is delocalized through the ring. The second lowest state is degenerate and has *p*-type orbitals, where there are two lobes along different axes. Examples of these solutions to the Schrödinger equation, using the discrete variable representation, are shown below in Supplementary Fig. 6. Due to the continuous rotational symmetry, these solutions are not unique. A rotation of any amount of the *p*-type orbitals would also be a solution. A perfect ring of this type could be excited by linearly polarized light along any in-plane direction, as there are valid *p*-type solutions for any rotation.

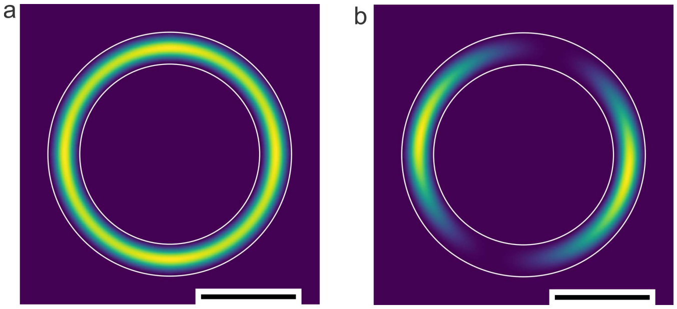

Supplementary Figure 6. Calculated wavefunctions of two-dimensional infinite ring well potentials with $OD_1 = OD_2 = 13$ nm, $ID_1 = ID_2 = 9.6$ nm. a, The ground state; b, The degenerate second lowest state. Scale bars: 5 nm.

Our theoretical modelling of the thin CdSe QRs using the empirical tight-binding method shows that the three-dimensional, atomistic ring structures adds additional complexity to their electronic structures. The discrete nature of the atomic positions breaks the continuous rotational symmetry present in the infinite ring potential, even in the case of $OD_1 = OD_2$ and $ID_1 = ID_2$, and this effect is more apparent compared to that observed in thicker QRs (see Fig. 4 in main text and Supplementary Notes 5 below). The exact effect of this broken symmetry depends on the specific cut of the ring out of the bulk. Displacing the origin of the supercell slightly results in different edges. Supplementary Fig. 7 shows the conduction band minima and valence band maxima for three different displacements of the origin, where we used $OD_1 = OD_2 =13$ nm and $ID_1 = ID_2 = 9.6$ nm. For some of the displacements, the *s*-type orbital nature of the conduction band minimum is still present, but for others, the wavefunctions are highly localized.

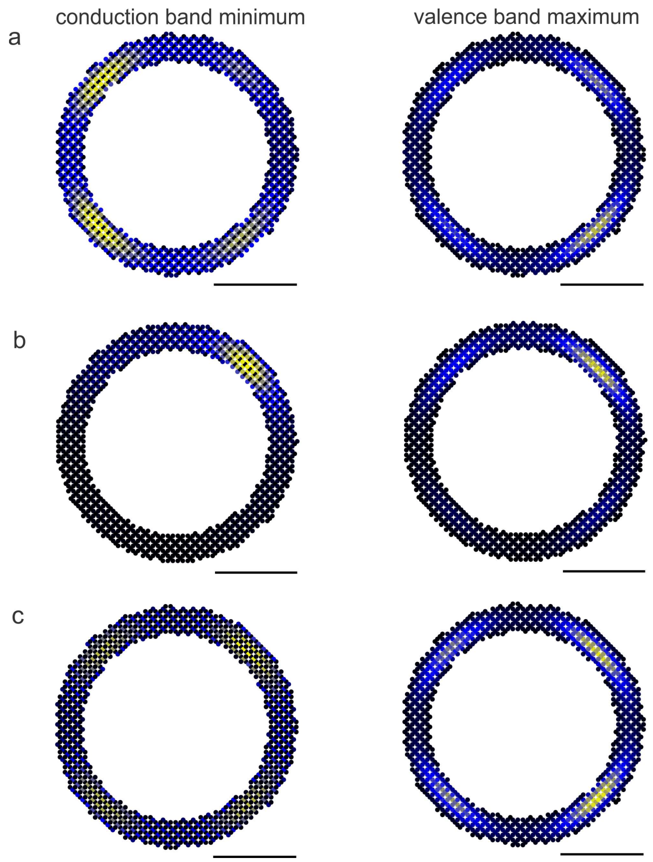

Supplementary Figure 7. Wavefunctions of conduction band minima (left) and valence band maxima (right) for quantum rings ($OD_1 = OD_2 = 13$ nm, $ID_1 = ID_2 = 9.6$ nm) with three different origins. Scale bars: 5 nm.

For CdSe quantum rings with $OD_1 \neq OD_2$, both the oval nature of the quantum ring and the atomic structure break rotational symmetry. The oval shape of the ring sets natural locations of the lobes; the local atomic structure of those locations changes the relative energy of each of the lobes. This leads to a localization of the band edge states into individual lobes. Examples are shown in Supplementary Fig. 8, where we used $OD_1 = 13$ nm, $OD_2 = 10$ nm, $ID_1 = 9.6$ nm, $ID_2 = 6.6$ nm, and various displacements of the origin. Consequently, optical transition dipole moments between these band-edge states will be uniaxial and in-plane.

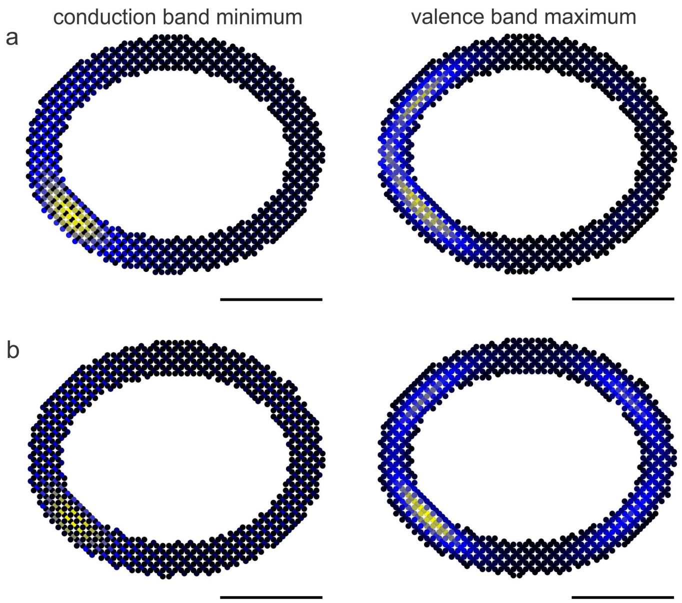

Supplementary Figure 8. Examples of conduction band minimum (left) and the corresponding valence band maximum (right) of CdSe rings with $OD_1 = 13$ nm, $OD_2 = 10$ nm, $ID_1 = 9.6$ nm, $ID_2 = 6.6$ nm with different displacements. Scale bars: 5 nm.

Supplementary Note 5 - Wavefunctions of thick CdSe quantum rings

Effects of the local atomic structures are almost negligible in thick CdSe quantum rings (Supplementary Fig. 9). In this case, only the oval geometry and the different ring thicknesses break the rotational symmetry and define the wavefunction localization. Both the electron and hole wavefunctions are localized in the thick end of the ring. Optical transition dipoles defined by these two states are highly uniaxial.

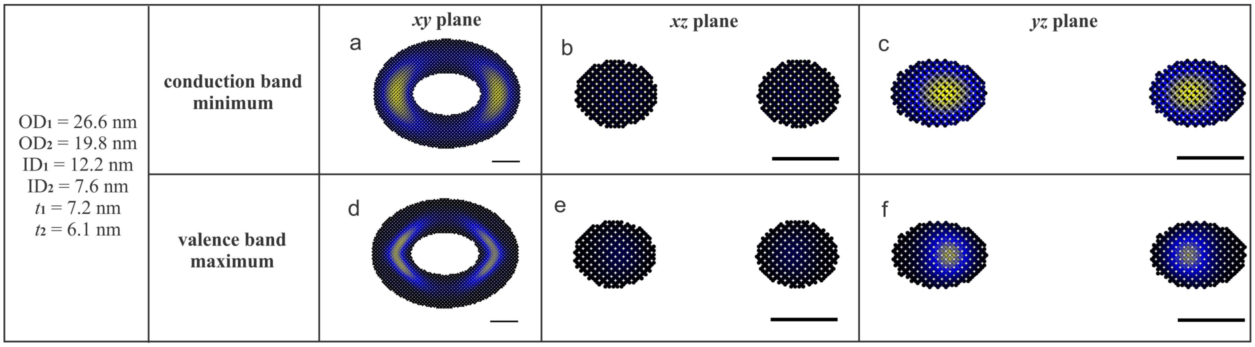

Supplementary Figure 9. 2D slices of wavefunctions of a thick CdSe QR along different directions. All the slices go through the center of the rings. Scale bars: 5 nm.

Supplementary Note 6 - Crystallographic dependence of the QR wavefunctions

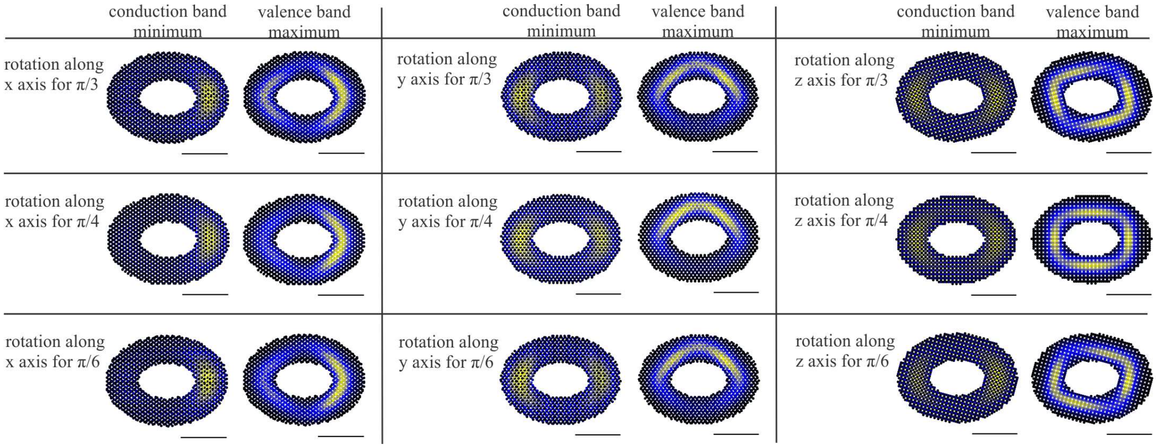

Supplementary Figure 10. Influences of crystallographic orientations on the wavefunctions of the QRs. Aside from the crystallographic orientation, the other parameters of the QRs are the same as those simulated in the main text. Scale bars: 5 nm.

Supplementary Note 7 - Inner hole dependence of the QR wavefunctions

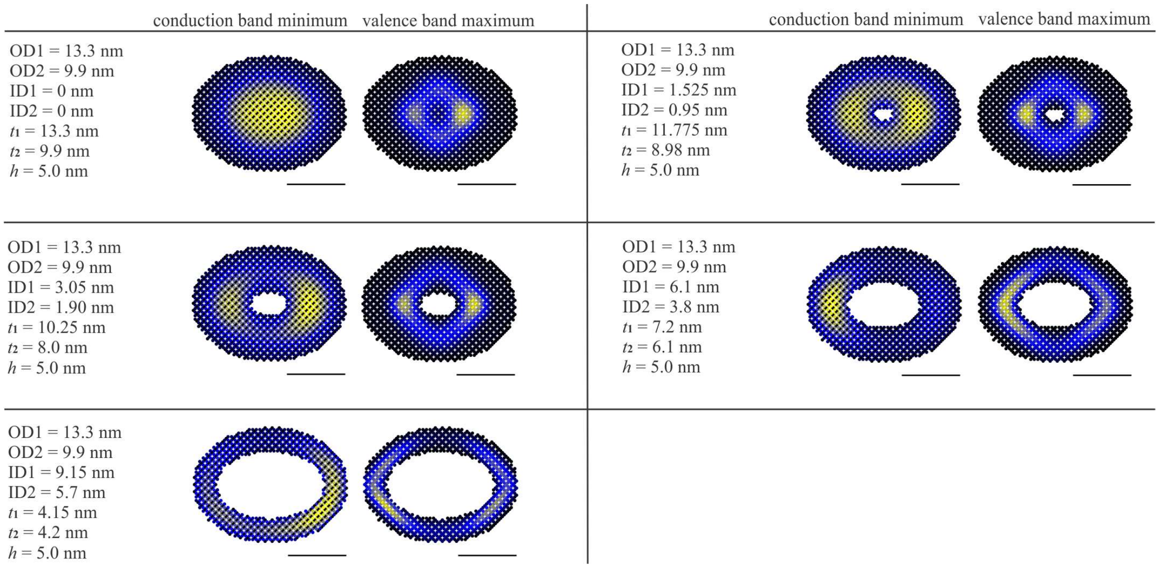

Supplementary Figure 11. Wavefunctions of QRs with their OD_1 and OD_2 kept constant, and ID_1, ID_2, t_1, and t_2 scaled proportionally. Scale bars: 5 nm.

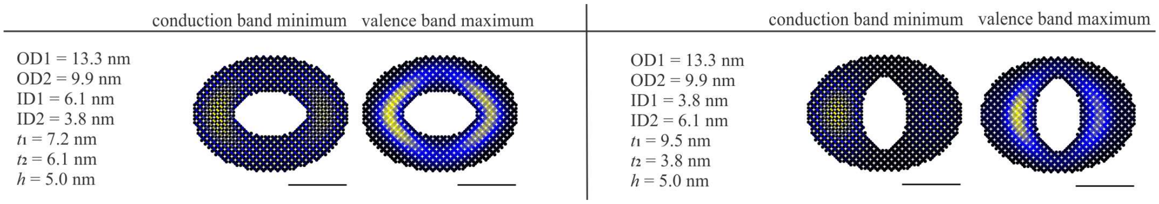

Supplementary Figure 12: Wavefunctions of QRs with (right) and without (left) inverted aspect ratios of their inner ovals. Scale bars: 5 nm. Inverting the aspect ratio of their inner oval (right) creates much larger regions for the wavefunctions to localize, nearly creating two separate quantum dots. There is still some anisotropy, but it is not nearly as strong.

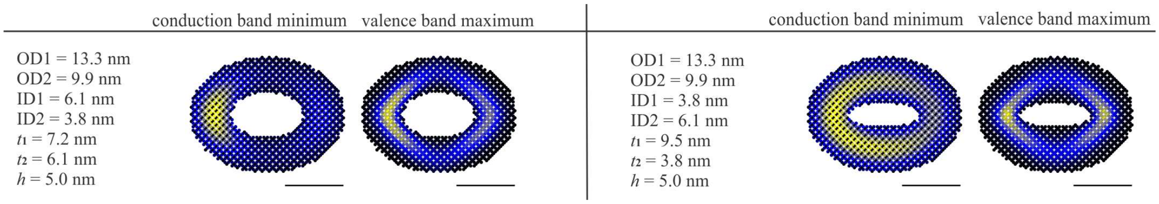

Supplementary Figure 13: Wavefunctions of QRs with the thicker end along the long (left) and short (right) axes. Scale bars: 5 nm. Having the thicker end along the short axis direction allows the wavefunctions to be more spread out and destroys the uniaxial dipole moment, almost returning the circular symmetry of the conduction band edge present in the perfect ring structure (right column). The thicker part of the ring allows for more wavefunction localization while the elongation causes a preferred localization along the edges. These two effects, at least in some geometries, counteract to return wavefunctions distributed throughout the ring, even with a lack of circular symmetry (right column).

Supplementary References:

1. Kurvits, J. A., Jiang, M. & Zia, R. Comparative analysis of imaging configurations and objectives for Fourier microscopy. *J. Opt. Soc. Am A* **32**, 2082-2092 (2015).

2. Lieb, M. A., Zavislan, J. M. & Novotny, L. Single-molecule orientations determined by direct emission pattern imaging. *J. Opt. Soc. Am. B* **21**, 1210-1215 (2004).
